# Design, Characterization, and Enhanced Performance of Electrospun Chitosan-Based Nanocomposites Reinforced with Halloysite Nanotubes and Cerium Oxide Nanoparticles for Wound Healing Applications

**DOI:** 10.3390/ijms262110520

**Published:** 2025-10-29

**Authors:** Valentina A. Petrova, Natallia V. Dubashynskaya, Sergei G. Zhuravskii, Daria N. Poshina, Alexey S. Golovkin, Alexander I. Mishanin, Iosif V. Gofman, Elena M. Ivan’kova, Maria Y. Naumenko, Galina Y. Yukina, Elena G. Sukhorukova, Arina D. Filippova, Vladimir K. Ivanov, Alexander V. Yakimansky, Yury A. Skorik

**Affiliations:** 1 Branch of Petersburg Nuclear Physics Institute named by B.P. Konstantinov of National Research Centre «Kurchatov Institute»-Institute of Macromolecular Compounds, Bolshoi VO 31, St. Petersburg 199004, Russia; 2 Hearing and Speech Laboratory, Pavlov First Saint Petersburg State Medical University, L’va Tolstogo 6-8, St. Petersburg 197022, Russia; 3 Almazov National Medical Research Centre, Akkuratova 2, St. Petersburg 197341, Russia; 4 Laboratory of Pathomorphology, Pavlov First Saint Petersburg State Medical University, L’va Tolstogo 6-8, St. Petersburg 197022, Russia; 5 Kurnakov Institute of General and Inorganic Chemistry, Russian Academy of Sciences, Leninskii 31, Moscow 119071, Russia

**Keywords:** chitosan, halloysite nanotubes, cerium oxide nanoparticles, electrospun scaffolds, wound healing, biocompatibility

## Abstract

The development of advanced wound dressings that integrate favorable physico-mechanical properties with the ability to support physiological healing processes remains a critical challenge in biomaterials science. An ideal dressing should modulate the wound microenvironment, prevent infection, maintain hydration, and possess adequate strength and elasticity. This study aimed to fabricate and characterize electrospun chitosan (CS)-based 3D scaffolds dual-reinforced with halloysite nanotubes (HNTs) and cerium oxide nanoparticles (CeONPs) to enhance material properties and biological performance. HNTs were incorporated to improve electrospinnability and provide mechanical reinforcement, while CeONPs were added for their redox-modulating and anti-inflammatory activities. Composite mats were fabricated via non-capillary electrospinning. The individual and synergistic effects of HNTs and CeONPs were systematically evaluated using physico-chemical methods (SEM, EDX, WAXS, TGA, mechanical testing) and biological assays (in vitro cytocompatibility with mesenchymal stem cells, in vivo biocompatibility, and wound healing efficacy in a rat model). Scaffolds containing only HNTs exhibited defect-free nanofibers with an average diameter of 151 nm, whereas the dual-filler (CS-PEO-HNT-CeONP) composites showed less uniform fibers with a rough surface and a larger average diameter of 233 nm. The dual-filler system demonstrated significantly enhanced mechanical properties, with a Young’s modulus nearly double that of pure CS mats (881 MPa vs. 455 MPa), attributed to strong interfacial interactions. In vivo, the CS-PEO-HNT-CeONP scaffolds degraded more slowly, promoted earlier formation of a connective tissue capsule, and elicited a reduced inflammatory response compared to single-filler systems. Although epithelialization was temporarily delayed, the dual-filler composite ultimately facilitated superior tissue regeneration, characterized by a more organized, native-like collagen architecture. The synergistic combination of HNTs and CeONPs within a CS matrix yields a highly promising scaffold for wound management, offering a unique blend of tailored biodegradability, enhanced mechanical strength, and the ability to guide healing towards a regenerative rather than a fibrotic outcome, particularly for burns and traumatic injuries.

## 1. Introduction

Human skin serves as a critical barrier against a variety of external threats, including biological pathogens such as bacteria. Consequently, any damage to the skin must be repaired promptly and effectively to restore its protective function [1]. Wound healing is a complex, multi-step process involving three overlapping stages: inflammation, new tissue formation and remodeling [2]. To develop advanced wound dressings, it is essential to consider the physiological and biochemical processes that characterize each phase of healing.

When a blood vessel is injured, the coagulation cascade is activated, resulting in the formation of a blood clot composed of cross-linked fibrin fibers and extracellular matrix (ECM) proteins [3]. At the same time, coagulation factors initiate an inflammatory response by activating the complement system, recruiting neutrophils and monocytes to the site of injury, and releasing cytokines and growth factors from activated platelets [4]. As inflammation progresses, the second phase of wound healing begins, characterized by the synthesis of ECM components such as type III collagen, proteoglycans, glycosaminoglycans (including hyaluronic acid), and the initiation of re-epithelialization and granulation tissue formation [1,5,6,7,8,9]. The final phase, tissue remodeling, involves a shift in ECM composition toward stronger and stiffer molecules, such as the replacement of type III collagen with type I collagen and a reduction in hyaluronic acid content [10,11]. This phase results in the formation of scar tissue, which is structurally similar to normal skin but often has reduced elasticity [12]. Although scarring is a natural consequence of wound healing, pathological conditions such as hypertrophic scars and keloids can occur and may require targeted treatment [1,13,14].

The wound healing process can be enhanced by the use of polymeric biomaterials that interact with tissues and modulate cellular processes during inflammation, tissue formation, and remodeling [15]. Different types of wounds—such as mechanical, thermal, chemical or trophic injuries; acute or chronic; superficial or full-thickness; aseptic or infected—have unique healing requirements. However, common factors influencing healing include oxygenation (critical for oxidative destruction of pathogens and compromised in chronic hypoxia), bacterial infection, and maintenance of a moist wound environment (which promotes cell proliferation, epithelialization, and reduces scarring and pain) [16,17]. An ideal wound dressing should address these physiological and biochemical factors, minimize pain, prevent microbial contamination, promote healing and improve patient quality of life. In addition, occlusive dressings that seal the wound and prevent dehydration are beneficial [15,18]. Furthermore, dressings should have adequate strength and elasticity [19].

Among modern wound dressings, 3D electrospun materials based on polysaccharides such as chitin, chitosan (CS), hyaluronic acid, cellulose, and alginate are particularly promising [20,21,22,23]. These materials are biocompatible, semipermeable to water vapor, highly absorbent, and capable of stimulating granulation and epithelialization while exhibiting antimicrobial activity. Their 3D structure also mimics the native ECM, making them highly effective for wound healing [24,25,26,27].

CS, a polymer of natural origin, is an excellent candidate for wound dressings due to its non-toxicity, biocompatibility, and antimicrobial activity against both Gram-positive and Gram-negative bacteria [28]. CS can be easily processed into electrospun materials from dilute acetic acid solutions, and bioactive compounds such as antimicrobial agents can be incorporated into its 3D structure [29]. However, the application of CS is constrained by two principal limitations: the inherently poor mechanical properties of CS-based matrices and challenges associated with electrospinning. The electrospinnability of CS is hampered by its polycationic nature, rigid chain structure, and pronounced inter- and intramolecular interactions, which often lead to the formation of beads or nanoparticles rather than continuous nanofibers [30,31]. A common strategy to overcome these issues involves blending CS with synthetic co-spinning polymers, such as polyvinyl alcohol [32], polylactic acid [33], and polyethylene oxide (PEO) [34], which enhances both spinnability and mechanical strength [35]. The physical-mechanical properties and morphology of the resulting composite nanofibers are highly dependent on the content of the synthetic polymer, which typically ranges from 20 to 90 wt%. To maximize the retention of CS’s beneficial biological properties, minimizing the amount of the synthetic additive is preferable [31]. In this context, PEO is particularly effective; its macromolecules form strong hydrogen bonds with the rigid CS chains. This interaction reduces the density of protonated amino groups in CS, thereby facilitating electrospinning and enabling the formation of continuous fibers even at low PEO concentrations of 5–10% [31,36]. In addition, the incorporation of nanofillers into CS matrices can yield composites with tunable properties, including improved mechanical strength, electrical conductivity, thermostability, and bioactivity [37]. For example, CS matrices can be reinforced with metal nanoparticles, metal oxides, or aluminosilicate clay minerals [38,39].

Halloysite hydrosilicate nanotubes (HNTs) are highly attractive as nanofillers due to their high aspect ratio, exceptional mechanical strength, and low density [40]. Furthermore, HNTs exhibit notable biocompatibility, low toxicity, and the ability to be cleared from the body via macrophage-mediated endocytosis [41]. Structurally, HNTs typically measure 500–2000 nm in length, with inner and outer diameters ranging from 10–30 nm and 30–50 nm, respectively. The outer surface of HNTs consists of silicon oxide, while the inner lumen is composed of aluminum oxide, resulting in a strongly negative ζ-potential (~−30 mV) on the outer surface and a positive charge (~+25 mV) within the inner cavity. This charge distribution enables HNTs to interact effectively with cationic polymers, such as CS, thereby enhancing their functional properties. Due to these characteristics, HNTs have been widely employed as reinforcing additives in the fabrication of CS-based matrices for tissue engineering applications [42,43,44]. For example, Devi and Dutta [45] developed ternary nanocomposite films using CS, starch, and HNTs, demonstrating significant improvements in water retention capacity, mechanical strength, and hemocompatibility. Similarly, De Silva et al. [46] incorporated HNTs into alginate nanofibers, enhancing their tensile strength and thermal stability. Additionally, Liu et al. [42] fabricated a highly porous CS-HNT composite sponge with superior mechanical and thermal properties while maintaining excellent cytocompatibility. Furthermore, HNTs are increasingly utilized in advanced wound dressings to promote healing in various wound types, such as burns, chronic wounds, and diabetic foot ulcers. This application is driven by their high mechanical strength, excellent biocompatibility, hemostatic efficacy, and demonstrated ability to enhance wound healing by accelerating re-epithelialization and collagen deposition. For example, a study by Liu et al. [47] reported that composite CS-HNT sponges exhibited a 9-fold increase in blood clotting activity compared to pure CS sponges. Moreover, in an in vivo evaluation using a full-thickness excisional wound model in Sprague Dawley rats, the CS-HNT composites significantly accelerated the healing process, demonstrating a 3 to 21-fold higher wound closure ratio than pure CS after one week.

Cerium oxide nanoparticles (CeONPs) represent another highly promising filler for wound dressing applications, owing to their unique redox activity that imparts potent antioxidant, anti-inflammatory, and antibacterial properties [48,49,50]. These nanoparticles exhibit significant anti-inflammatory effects in engineered tissues through multiple mechanisms, including reactive oxygen species scavenging, inflammation suppression, cytokine level modulation, and cellular protection [51,52,53]. Numerous studies have demonstrated the protective role of CeONPs against oxidative stress and inflammatory responses across various mammalian cell types [54,55,56]. When incorporated into CS matrices, CeONPs not only enhance mechanical properties but also significantly improve cytocompatibility and accelerate wound healing, even in challenging cases such as chronic diabetic wounds [57,58,59,60]. For example, Naseri-Nosar et al. [61] developed a porous wound dressing composed of poly(ε-caprolactone)/gelatin electrospun mats incorporating 1.5% CeONPs. This construct exhibited favorable characteristics, including wettability, mechanical strength, water absorption capacity, and water vapor transmission rate. In an in vivo evaluation using Wistar rats, the CeONP-containing dressing achieved complete wound closure (100%) within two weeks, a significant enhancement compared to the 63% closure observed with the CeONP-free control. Another notable study by Sener et al. [62] fabricated zwitterionic cryogels from [2-(methacryloyloxy)ethyl]dimethyl-(3-sulfopropyl)ammonium hydroxide, 3-[[2-(methacryloyloxy)ethyl]dimethylammonio]propionate, and 2-hydroxyethyl methacrylate, which were co-loaded with CeONPs and microRNA. These systems demonstrated significant efficacy in a diabetic mouse wound healing model, an effect attributed to the ability of CeONPs to scavenge reactive oxygen species and reduce oxidative stress.

In our previous study [36], we developed electrospun CS fiber mats incorporating CeONPs, which exhibited reduced fiber diameter and increased Young’s modulus compared to pure CS mats. However, these composites displayed a tendency to crumple in surgical implant pockets, occasionally leading to aseptic reactive inflammation during biodegradation. The integration of nanoceria into polymer composites enables a synergistic combination of the advantageous properties of both components. This approach enhances the biomedical potential of CeONPs by addressing critical limitations such as nanoceria solubility and uncontrolled cerium ion release, thereby reducing potential toxicity [63]. Concurrently, the polymer matrix benefits from improved biomolecule and cell adhesion, along with enhanced overall biocompatibility.

While the individual merits of HNTs and CeONPs in composite materials are established, their combined effect is not merely additive. We hypothesize that their incorporation into a CS matrix will yield a synergistic effect, resulting in a scaffold with properties superior to those achieved by either filler alone. Mechanistically, we propose that the rigid HNTs will provide primary structural reinforcement by forming a network within the fibrous matrix, thereby significantly enhancing the mechanical strength and thermal stability. Simultaneously, the redox-active CeONPs are expected to not only improve cytocompatibility but also to chemically interact with the polymer and HNTs. Specifically, the CeONPs may act as cross-linking points, further stabilizing the polymer matrix and modulating its degradation rate. This unique combination is anticipated to create a more balanced microenvironment for tissue regeneration in damaged integument: the HNT-reinforced scaffold maintains structural integrity longer, while the CeONPs reduce oxidative stress and inflammation, ultimately guiding the granulation tissue and guide the healing process toward a more physiological outcome, minimizing the potential for excessive scar formation. The primary objective of this study is to develop an electrospun material composed of CS, reinforced with HNTs and CeONPs. This innovative approach is expected to yield 3D polymeric scaffolds exhibiting enhanced mechanical properties and superior biocompatibility. These characteristics make the proposed material a promising candidate for effective management of a broad spectrum of wound types.

## 2. Results and Discussion

### 2.1. Fabrication of Electrospun Biohybrid Scaffolds (CS-PEO-HNT and CS-PEO-HNT-CeONP)

**Figure 1:**
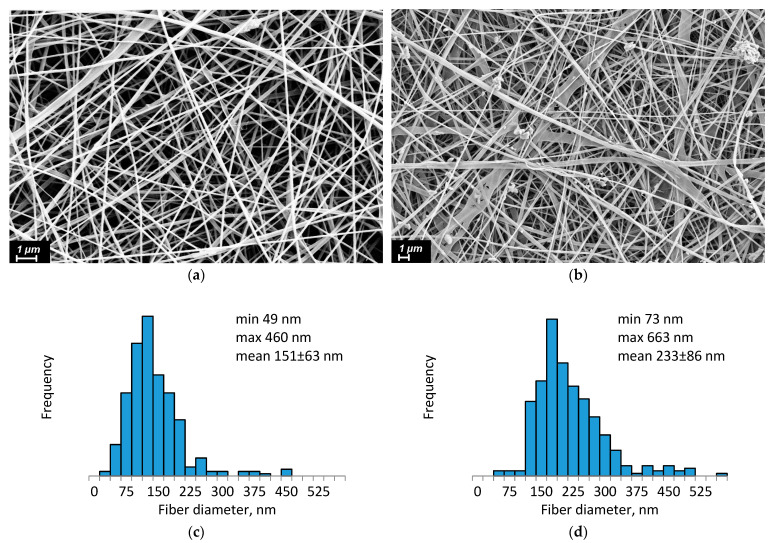
SEM images and fiber diameter distribution of CS-PEO-HNT (**a**,**c**) and CS-PEO-HNT-CeONP (**b**,**d**) nanofibers.

The electrospun 3D scaffolds exhibit a high surface area, excellent water and gas permeability, and a structure that closely mimics ECM of body tissues, making them highly promising for wound healing applications [64]. After preparation, the CS-PEO-HNT and CS-PEO-HNT-CeONP electrospun mats were heated at 80 °C for 3 h to remove unbound acetic acid and convert CS into its water-insoluble form. SEM revealed the uniform formation of nanofibers in both mats. The composite solution containing HNTs produced smooth, defect-free nanofibers (Figure 1a), with an average fiber diameter of approximately 151 ± 63 nm (Figure 1c).

In contrast, the morphology of composite nanofibers containing both HNTs and CeONPs showed less homogeneity. Individual nanofibers exhibited a rough surface, likely due to the aggregation of different nanoparticles on the fiber surface (Figure 1b). The average diameter of these fibers was larger, measuring 233 ± 86 nm (Figure 1d).

**Figure 2:**
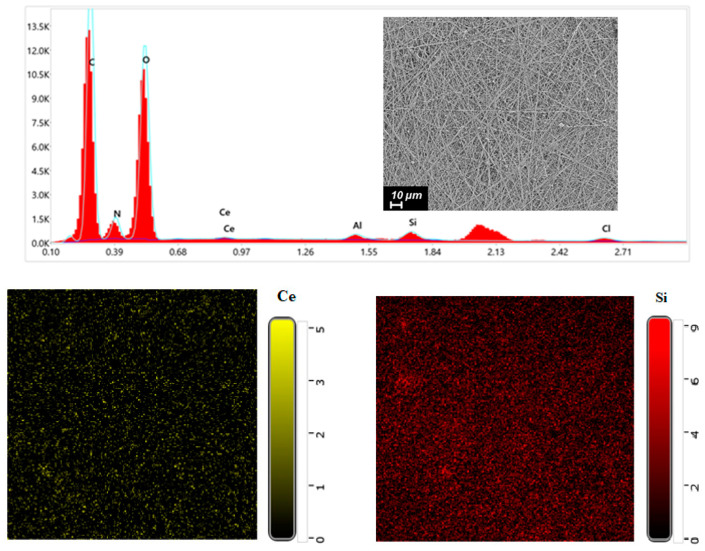
EDX spectrum and elemental distribution maps for CS-PEO-HNT-CeONP nanofibers.

EDX was performed on CS-PEO-HNT-CeONP to confirm the presence of CeONPs (Ce) and HNTs (Si) within the nanofibers (Figure 2). Elemental distribution maps indicated a uniform dispersion of HNTs and CeONPs throughout the electrospun material.

### 2.2. Swelling Properties

CS interacts with the surface of HNTs, and this interaction can be enhanced through heating, as previously noted [45]. The incorporation of HNTs resulted in a reduction in the swelling degree of the CS-PEO-HNT electrospun mats compared to the CS-PEO material (Table 1). Conversely, the addition of CeONPs led to an increase in the swelling degree of the CS-PEO-CeONP composite in both water and 0.9% NaCl solution (Table 1), attributed to the rearrangement of intermolecular bonds within the CeONP system. Similar swelling properties were previously observed in CS-PEO-CeONP composites (Table 1, [36]). Additionally, we have reported similar results for CS-based films containing CeONPs, which exhibited increased hydrophilicity compared to pure CS films [57].

### 2.3. Wide-Angle X-Ray Scattering (WAXS)

The WAXS of the CS-PEO-HNT-CeONP electrospun composite was quite challenging due to the low mass content of HNTs and CeONPs and the high porosity of the sample. The halloysite peaks overlapped with those of CS and PEO and appeared as a shoulder at 2θ of 12°. Weak reflections of CeONPs were observed at 2θ values of 28.7°, 33.0°, 47.5°, and 57.0°, corresponding to the (110), (200), (220), and (311) crystal planes of CeO_2_ (fluorite cubic crystal structure), respectively: ICDD PDF Map #34-394, data from National Institute of Standards and Technology, Gaithersburg, MD, USA.

**Figure 3:**
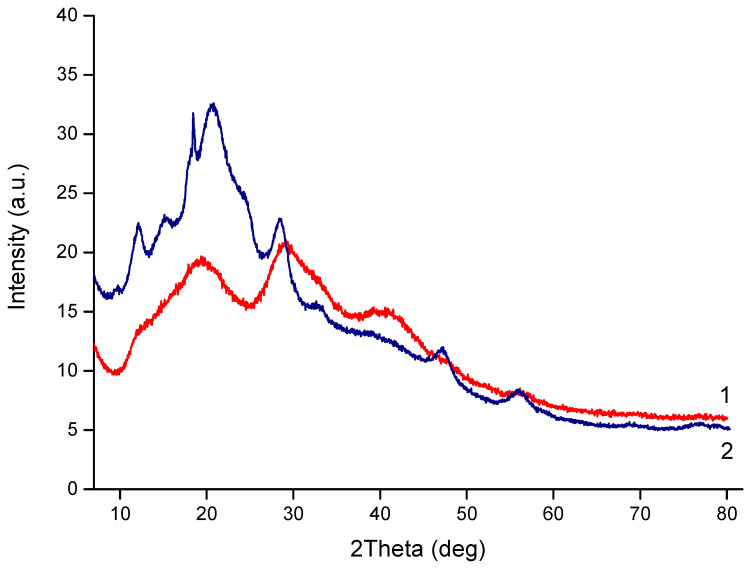
XRD profiles of CS-PEO-HNT-CeONP electrospun mat (1) and CS-PEO-HNT-CeONP film (2).

More intense reflections characteristic of CeONPs were observed in a CS-PEO-HNT-CeONP film of similar composition (Figure 3). This suggests that the morphology of the porous nonwoven composite influences the X-ray diffraction analysis. We have previously observed similar effects when comparing the diffractograms of multilayer composites in film and nonwoven forms [65].

### 2.4. Mechanical Properties

**Figure 4:**
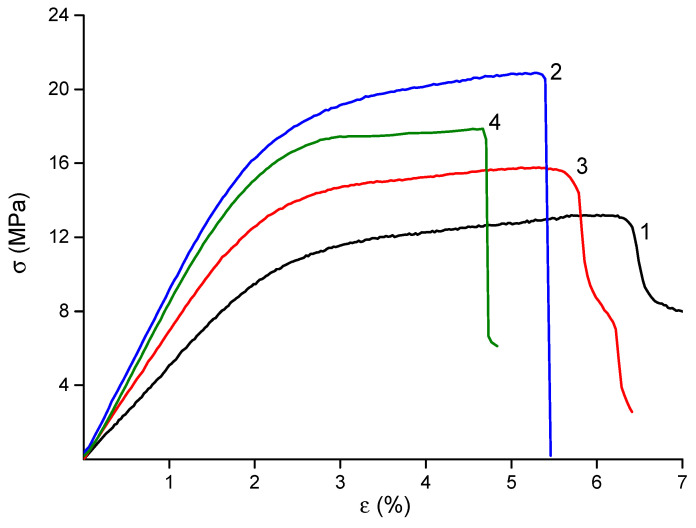
Stress–strain curves of CS-PEO [36] (1), CS-PEO-HNT (2), CS-PEO-CeONP [36] (3) and CS-PEO-HNT-CeONP (4) electrospun mats.

The mechanical properties of the materials are summarized in Table 2, and their stress–strain curves are shown in Figure 4. The deformation behavior of all samples was typical of electrospun materials, with no necking observed beyond the yield point. The final strain values were in the range of 5–6%, which is suitable for biomedical applications.

The incorporation of nanofillers into the CS polymer matrix resulted in materials with enhanced mechanical properties. The observed near-doubling of the Young’s modulus in the dual-filler composite (CS-PEO-HNT-CeONP) compared to the pure CS-PEO mat supports our hypothesis of a synergistic reinforcing effect, where the HNTs provide the primary structural support and the CeONPs potentially contribute to additional interfacial bonding. The high strength of the HNT-containing material is attributed to the reinforcement of the polymer matrix by rigid nanotubes and the interaction between positively charged CS and negatively charged HNTs (Table 2). The introduction of CeONPs also increased the stiffness of the material, albeit to a lesser extent, due to the formation of additional bonds between polymer macromolecules and nanoparticle surfaces [36,57]. However, the formation of nanocomposites led to a slight reduction in the final deformation of the samples (Table 2), a common phenomenon in polymer-inorganic nanocomposites [66]. The most significant reduction in ε_b_ was observed in the electrospun mat containing both HNTs and CeONPs.

### 2.5. Thermal Properties

**Figure 5:**
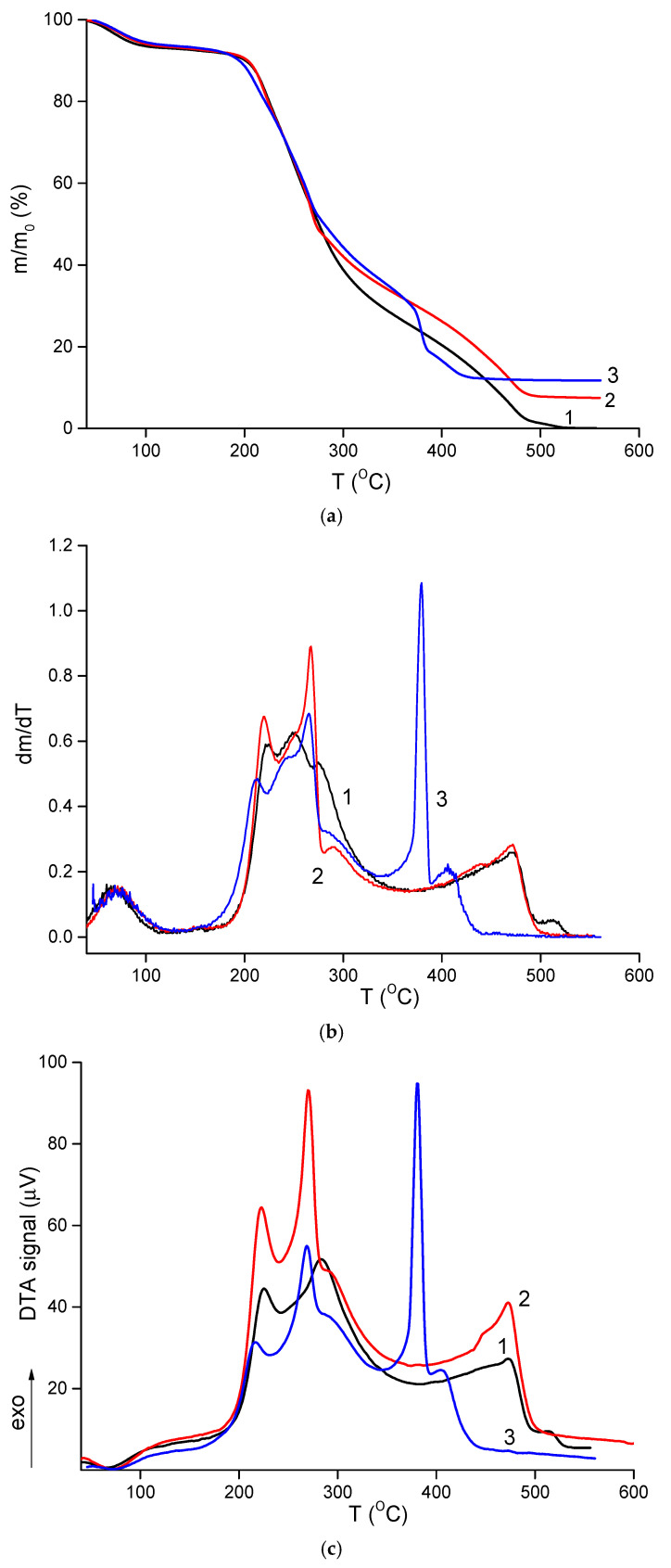
TGA (**a**), differential TGA (**b**), and DTA (**c**) curves of CS-PEO [36] (1), CS-PEO-HNT (2), and CS-PEO-HNT-CeONP (3) electrospun mats.

TGA results are shown in Figure 5a. All materials contained approximately 7% moisture, which evaporated between 120–130 °C. Thermal degradation of the composites began at 150–160 °C. Similar to other polysaccharide-based materials, these electrospun composites underwent thermo-oxidative degradation in two stages, as evident in the differential mass loss curves (Figure 5b). The first stage occurred between 170–300 °C, during which the samples lost about 50–55% of their initial weight. This stage involved multiple overlapping processes, with maximum intensity at 210–225 °C and 250–265 °C, reflecting the degradation characteristics of both polymers. No significant differences were observed between the thermal behavior of the CS-PEO mat and the HNT-containing nanocomposite (Table 3). However, the introduction of CeONPs shifted the degradation to lower temperatures, with t_5_ and t_10_ values decreasing by 8–10 °C (Table 3).

The second stage of intense degradation occurred above 360–370 °C. In this range, the CS-PEO-HNT-CeONP mat exhibited a more pronounced weight loss, with 12% of its initial weight lost between 365–385 °C. This was accompanied by a sharp exothermic peak in the DTA curve (Figure 5c), which was 90 °C lower and more intense compared to the other samples. This effect is attributed to the catalytic role of cerium oxide in the degradation of organic materials [67]. However, this catalytic effect is not critical for the biomedical application of the CS-PEO-HNT-CeONP composite, as it occurs during the deep degradation stage when the material is no longer functional. The reduced thermal stability of the composite fibers containing both HNTs and CeONPs aligns with mechanical and morphological results, suggesting that nanoparticle interactions increase the heterogeneity of the nanofibers.

### 2.6. Cytocompatibility Study

The cytocompatibility of the fabricated electrospun mats was evaluated using mesenchymal stem cells (MSCs) as a model system, with particular emphasis on cellular adhesion and proliferation dynamics. MSCs were selected as a representative cell type for assessing potential cytotoxic effects relevant to various somatic cell populations. Quantitative analysis revealed a statistically significant reduction (*p* < 0.05) in MSC counts on CS-PEO-HNT-CeONP substrates compared to CS-PEO-HNT and to CS-PEO control (Table 4). Notably, MSC cultures on CS-PEO-HNT-CeONP matrices exhibited a distinct morphological response characterized by spontaneous spheroid formation, suggesting altered cell–substrate interactions.

**Figure 6:**
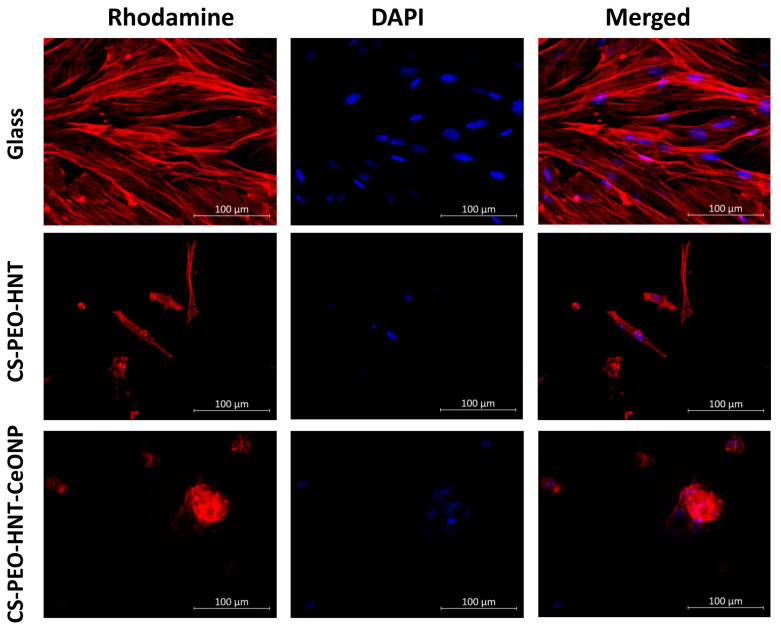
Representative microphotographs of MSCs adhered to the sample surface (×400 magnification). All experiments were performed in triplicate, with at least 10 random fields of view captured per replicate.

Cell morphology also differed between the samples. On CS-PEO-HNT, adhesive cells were abundant, with most exhibiting a typical elongated shape and longitudinal striations. However, some cells appeared spherical with protrusions. The elongated cells were smaller in area compared to those on coverslips, and cell colonies were not distinctly visible. In contrast, CS-PEO-HNT-CeONP displayed many single cells with a nearly rounded shape, with only a few elongated cells present. Numerous small spheroid colonies, measuring up to 66 ± 11 μm in diameter, were observed on the sample surface. Some spheroids exhibited signs of cell migration along the colony periphery (Table 4, Figure 6).

In summary, the developed biohybrid scaffolds exhibited satisfactory cytocompatibility profiles in vitro when evaluated using rat MSCs as a model system. Quantitative assessment revealed that the incorporation of dual nanofillers (CS-PEO-HNT-CeONP) resulted in moderately reduced cellular compatibility compared to the HNT-only formulation (CS-PEO-HNT), as demonstrated by decreased cell adhesion efficiency and proliferative capacity (*p* < 0.05). Importantly, both scaffold variants maintained non-cytotoxic characteristics throughout the evaluation period, suggesting their fundamental biocompatibility despite the observed differences in cellular responses.

### 2.7. In Vivo Biocompatibility Assessment

**Figure 7:**
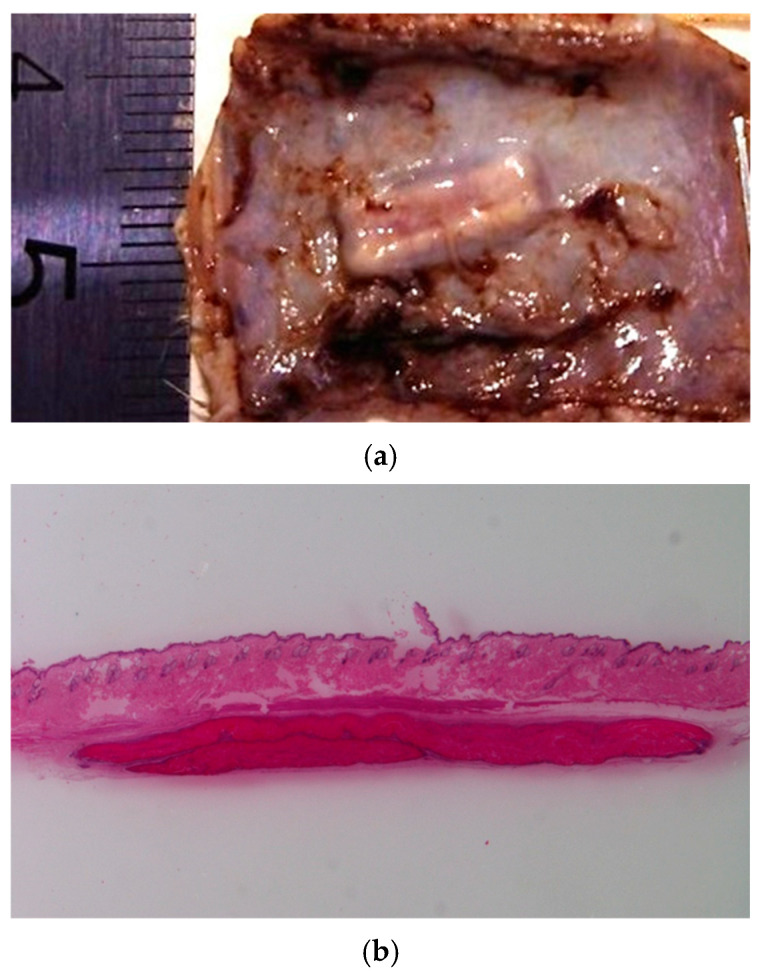
Images of the implanted CS-PEO-HNT-CeONP mat on day 30: formalin-fixed (**a**) and hematoxylin and eosin-stained cross-section (**b**), ×4 magnification. Visual inspection revealed no signs of surrounding scar tissue in either the formalin-fixed or the histological samples. Each group at each time point contained *n* = 3 biologically independent samples.

Subcutaneous implantation of the developed materials was performed to evaluate their in vivo biocompatibility and biodegradation. The results indicated uncomplicated wound healing in the absence of chronic pain, inflammation, or pronounced scar formation. The appearance and structure of the implanted CS-PEO-HNT-CeONP mat in a formalin-fixed specimen are shown in Figure 7a.

The CS-PEO-HNT mat in the implant pocket formed crumpled clumps up to 460 μm thick. Degradation began by the end of the first month, primarily through surface layer delamination. By the third month, the ratio of disintegrated to preserved mat was approximately 1:3 to 1:4. Single cells were visible within the mat, and a thin-walled connective tissue capsule without aseptic inflammation began to form.

**Figure 8:**
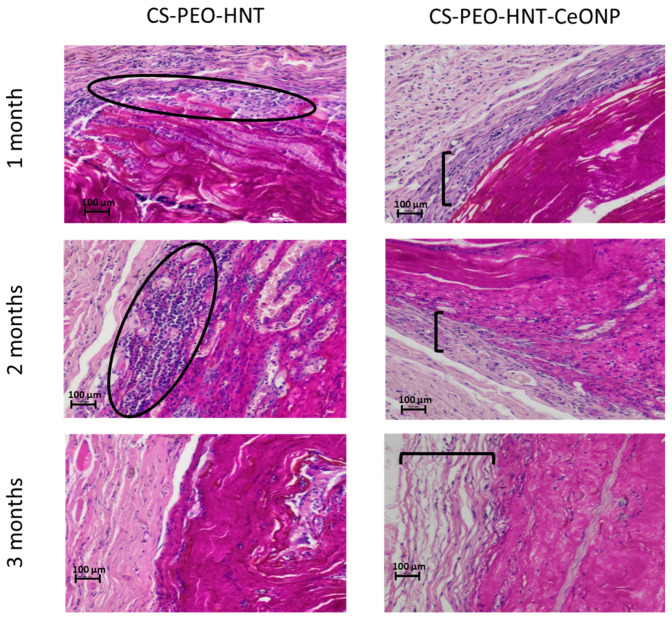
Histological patterns of the implantation site at different time intervals, ×100 magnification (hematoxylin and eosin staining). Ovals highlight zones of aseptic inflammation; brackets indicate the connective tissue capsule layer. Each group at each time point contained *n* = 3 biologically independent samples.

In contrast, CS-PEO-HNT-CeONP remained intact in the implant pocket without clumping, as shown in macroscopic images of formalin-fixed samples 30 days post-surgery (Figure 7). Histological analysis of the implantation site at various time intervals revealed that the dual-filler CS-PEO-HNT-CeONP mat degraded at a slower rate compared to CS-PEO-HNT. By the end of the first month, the mat remained dense, with no peripheral delamination. A key feature of its biocompatibility was the early formation of a connective tissue capsule. By the second month, the scaffold exhibited signs of degradation, accompanied by the formation of a well-defined connective tissue capsule with evident vascularization and a reduction in aseptic inflammation when compared to CS-PEO-HNT. By the third month, the mat thickness was 500–700 µm, with surface degradation occurring at approximately ¼ of its thickness. Inflammatory infiltration was largely absent, persisting only in areas of higher material disintegration. A thin, well-organized connective tissue capsule composed of collagen fibers was evident, with no signs of infiltration. The histological patterns of the implantation site at different time intervals are shown in Figure 8.

In summary, histological analysis confirmed the superior in vivo performance of the dual-filler composite. The CS-PEO-HNT-CeONP scaffold exhibited a slower degradation rate, superior structural integrity without clumping, and a significantly attenuated inflammatory response—as qualitatively assessed by histology—compared to the HNT-only formulation. Critically, these findings align with our initial mechanistic hypothesis: the structural reinforcement provided by HNTs, combined with the anti-inflammatory and proposed matrix-stabilizing effect of CeONPs, creates a more controlled and biocompatible degradation profile. This synergy promotes the early formation of a well-organized connective tissue capsule. While these qualitative results are highly promising, future studies incorporating quantitative analyses—such as immunohistochemical cell counts (e.g., for CD68+ macrophages) or cytokine profiling (e.g., TNF-α, IL-1β)—would provide robust objective evidence to further solidify these claims. Nonetheless, the present results underscore the potential of the CS-PEO-HNT-CeONP material as an effective scaffold for managing extensive skin defects, as it provides durable coverage while establishing an optimal regenerative microenvironment.

### 2.8. In Vivo Evaluation of Wound Healing

**Figure 9:**
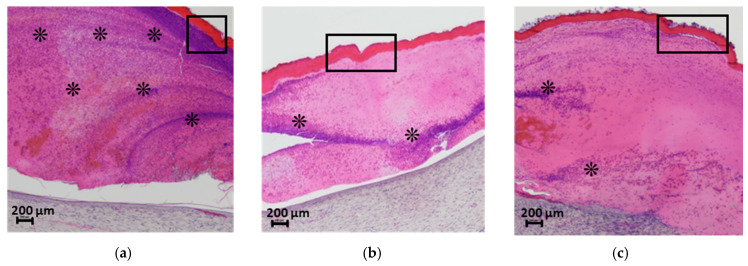
Histological analysis of the wound bed and implanted materials at 21 days post-surgery. Cross-sections of (**a**) CS-PEO, (**b**) CS-PEO-HNT, and (**c**) CS-PEO-HNT-CeONP scaffolds stained with hematoxylin and eosin (×40 magnification). The rectangular outlines highlight the scaffold regions, and the asterisks (*) indicate sites of cellular infiltration in the scab. Each group contained *n* = 2 biologically independent samples.

The wound-healing properties of the developed materials were assessed using a wound model that heals by primary intention (*per primam intentionem*). On the 21st day post-surgery, veterinary examinations of all animal groups revealed no spontaneous mortality or clinical signs of ill health, such as behavioral stress symptoms, abnormalities in coat or visible mucous membranes, or local complications like purulent infections, tissue necrosis, or contact allergic dermatitis. Over the 21-day observation period, neither the CS-PEO-HNT nor the CS-PEO-HNT-CeONP scaffolds underwent significant resorption. All samples remained sutured in place, albeit with varying degrees of adherence (Figure 9). By the end of the experimental period, wounds in the CS-PEO and CS-PEO-HNT groups exhibited complete scaffold detachment, which was facilitated by the presence of a newly regenerated epidermal layer. In contrast, wounds treated with CS-PEO-HNT-CeONP showed incomplete epithelial regeneration, and the scaffold consequently maintained persistent adhesion to the underlying granulation tissue.

**Figure 10:**
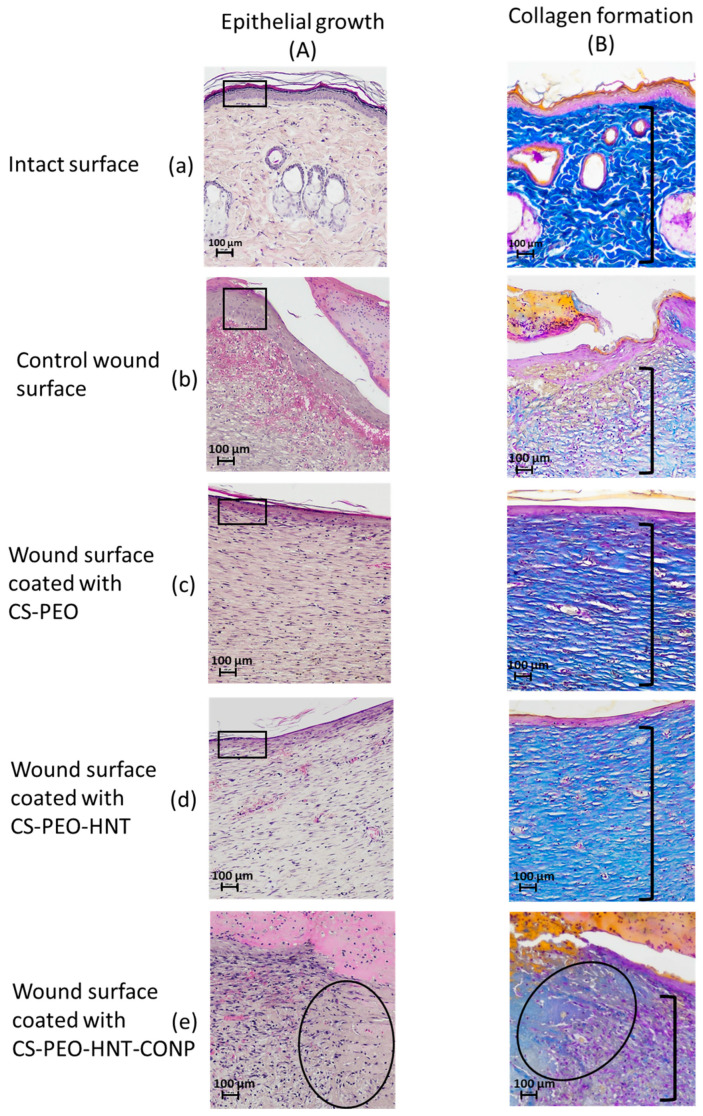
Wound healing assessment at 21 days post-surgery. Representative micrographs show epithelialization patterns (hematoxylin and eosin staining; (**A a–e**) and collagen deposition (Mallory’s staining; (**B a–e**) at ×100 magnification. In Group **A**, rectangles highlight the thickness of the newly formed epithelial layer, while an oval denotes areas of residual granulation tissue. In Group **B**, brackets indicate the dermal layer exhibiting the characteristic architecture of collagen fibers. Each group contained *n* = 2 biologically independent samples.

Histological analysis data are presented in Figure 10. Skin samples from the intact control animals (Figure 10B-a) clearly depict the well-defined collagen fibers characteristic of the reticular dermis. In the sham-operated control group, scab elements with minimal leukocyte infiltration and cellular debris were observed at 21 days post-surgery. The granulation tissue surface was covered by a continuous layer of stratified squamous epithelium, which was thicker compared to intact areas (Figure 10A-b). Histological sections of the skin flap revealed well-formed granulation tissue replacing the wound defect (Figure 10B-b), with extensive vascularization characterized by branching loops of engorged capillaries and vertical vessels. At this stage, signs of epidermal keratinization and papillary layer formation were evident. The granulation tissue consisted of abundant fibroblasts and thin collagen fibers arranged in a reticular pattern, with no signs of inflammation and a moderate presence of macrophages.

In the group treated with the CS-PEO mat, a thick scab heavily infiltrated with leukocytes and cellular debris was observed (Figure 9a). Epithelialization beneath the scab was complete, with the formation of a stratum corneum, although the epithelial layer was thinner than in intact areas and the control group (Figure 10A-c). The underlying layer exhibited dense, parallel bundles of collagen fibers without the formation of a three-dimensional mesh structure (Figure 10B-c). The cellular composition was predominantly fibroblasts, with few fibrocytes and macrophages. Vascularization was minimal, lacking loop-like vessels, and no inflammatory response was noted.

In the CS-PEO-HNT group, a substantial scab with mild leukocyte infiltration and cellular debris was present. The scab had completely detached from the skin surface (Figure 9b). The epithelial layer, though fully formed and covering the wound defect, was thinner than in intact areas (Figure 10A-d). Collagen fibers in the developing dermis were arranged in dense parallel bundles (Figure 10B-d), with fibroblasts being the predominant cell type and a moderate number of fibrocytes. A small number of macrophages were observed, but no inflammatory response was detected. Vascularization was moderate, without the formation of loop-like or vertical vessels.

In the CS-PEO-HNT-CeONP group, a thick scab with moderate leukocyte infiltration and cellular debris was observed. However, epithelial regeneration was incomplete, as a central area of the wound defect persisted, covered by the closely adhered scaffold (Figure 9c). At the periphery of the former wound, where epithelialization had occurred, the scaffold had detached. The tissue beneath the non-epithelialized central defect exhibited sparse, thin collagen fibers and signs of inflammation, including dilated thin-walled vessels (hyperemia), lymphocytes, and numerous active macrophages. Furthermore, in contrast to the other groups, the region beneath the newly formed epithelium contained fewer collagen fibers, which were arranged in a loose, reticulate pattern rather than the dense, parallel bundles seen elsewhere (Figure 10B-e). The cellular composition in this area included randomly distributed fibroblasts alongside a high density of active macrophages.

In summary, the comparative analysis revealed that the two composite scaffolds mediate distinct and mechanistically insightful wound healing patterns. The CS-PEO-HNT scaffold facilitated rapid epithelialization but resulted in a thinner epithelial layer and the formation of parallel collagen bundles—a histological signature of fibrotic healing and foreign body encapsulation.

In striking contrast, the CS-PEO-HNT-CeONP composite demonstrated a truly synergistic effect, orchestrating a fundamental trade-off between healing kinetics and tissue quality. While the initial epithelial migration was temporarily delayed, the ultimate outcome was the regeneration of a dermal architecture strikingly similar to native skin morphology. We propose that this occurs through a defined mechanistic framework: the HNTs provide sustained structural support, preventing scaffold collapse and enabling guided tissue formation, while the CeONPs attenuate the inflammatory response and modulate the oxidative microenvironment. Together, they create a pro-regenerative niche that promotes the development of an organized, reticular collagen structure instead of scar tissue.

This insight—that a delay in closure can be strategically exploited to achieve superior regeneration—opens a compelling avenue for future therapeutic design. It suggests the potential for a dual-phase treatment strategy, where an initial dressing promotes epithelialization, followed by the application of CS-PEO-HNT-CeONP to guide the remodeling phase and minimize scarring. Alternatively, a single, smart dressing could be engineered to temporally release bioactive factors, addressing both phases of healing simultaneously.

Consequently, these results robustly position the CS-PEO-HNT-CeONP composite not merely as a passive dressing, but as an active scaffold for advanced wound management, where the primary objectives are the quality of healing and scar reduction, moving beyond the paradigm of simply accelerating wound closure.

## 3. Materials and Methods

### 3.1. Materials

Crab CS (Bioprogress, Shchelkovo, Russia) with a characteristic viscosity ([η]) of 2.56 dL/g and a viscosity-average molecular weight (Mη) of 6.0 × 10^4^ was used in this study. The characteristic viscosity was determined in a solvent consisting of 0.33 M CH_3_COOH and 0.3 M NaCl using an Ubbelohde capillary viscometer at 20 °C. The molecular weight (Mη) was calculated using the Mark–Houwink equation: [η] = 3.41 × 10^−3^Mη^1.02^ [68]. The degree of deacetylation, determined by ^1^H NMR spectroscopy, was found to be 82%. PEO with a molecular weight of 9.0 × 10^5^ was purchased from Sigma-Aldrich (St. Louis, MO, USA), and HNTs were obtained from NaturalNano Inc. (Rochester, NY, USA). Citrate-stabilized CeONPs were synthesized following a previously reported procedure [69], yielding ultrasmall particles with a hydrodynamic diameter of approximately 2.3 nm (dynamic light scattering) and a core size of 1.5–2.5 nm (scanning electron microscopy, SEM), which form stable aqueous dispersions.

### 3.2. Electrospinning

To ensure homogeneous nanofiller distribution, both HNTs and CeONPs were subjected to pre-dispersion protocols. The CS-stabilized CeONP dispersion was prepared according to established methodology [57]. Briefly, a 1% aqueous dispersion of citrate-stabilized CeONPs was mixed with a 0.1% CS solution in 1% acetic acid using an IL 100-6 ultrasonic homogenizer (Ultrasonic Techniques-INLAB, St. Petersburg, Russia) for 10 min, maintaining a mass ratio of 1:1 between citrate-stabilized CeONPs and CS.

For HNT dispersion, the predetermined mass of nanotubes was initially hydrated in distilled water for 24 h, followed by 10 min of sonication using an IL 100-6 ultrasonic disperser (Ultrasonic Techniques-INLAB, St. Petersburg, Russia). This pre-dispersion was subsequently combined with a 0.2% CS solution in 1% acetic acid under continuous sonication.

The polymer solution was prepared using a CS concentration of 3%, previously established as optimal for achieving appropriate viscosity for stable jet formation in our non-capillary electrospinning configuration. CS powder was initially dispersed in deionized water under vigorous stirring for several hours, followed by addition of glacial acetic acid with continuous stirring until complete dissolution was achieved. A 3% aqueous PEO solution was then incorporated to yield the final spinning solution (CS-PEO solution) containing 3% CS and 0.3% PEO in 70% *v*/*v* acetic acid.

The HNT-containing spinning solution (CS-PEO-HNT solution) was formulated by introducing the pre-dispersed HNT suspension into the CS-PEO solution under vigorous stirring. The final composite solution (CS-PEO-HNT-CeONP solution) was prepared by sequential addition of pre-dispersed HNT and CeONP suspensions (5% HNTs and 8% CeONPs by weight of CS) to the CS-PEO solution under identical mixing conditions.

Electrospinning was performed using a non-capillary Nanospider NS Lab 500 apparatus (Elmarco, Liberec, Czech Republic) with an applied voltage of 50–65 kV to ensure stable jet formation. The spinning electrode rotation speed was maintained at 10 rpm with a fixed inter-electrode distance of 24 cm. Fibers were collected on a paper substrate, producing two distinct nanofiber mat variants: CS-PEO-HNT and CS-PEO-HNT-CeONP. Following electrospinning, all samples were conditioned at room temperature for 5 days followed by thermal treatment at 80 °C for 3 h. All electrospinning parameters, including polymer concentrations, solvent composition, applied voltage, inter-electrode distance, and collector rotation speed, were kept identical to those used in our previous study [36] to ensure a valid comparison of the resulting materials.

### 3.3. General Methods

The swelling capacity of the electrospun mats was evaluated gravimetrically by measuring their weight increase after immersion in both distilled water and physiological solution (0.9% NaCl). Precisely weighed air-dry samples (W_dry_) were immersed in the respective solutions at room temperature (20 ± 2 °C) until swelling equilibrium was reached. The swollen mats were then carefully removed, excess surface liquid was blotted away using filter paper, and they were immediately weighed again (W_wet_). The swelling degree was calculated according to the formula: Swelling (g/g) = [(W_wet_ − W_dry_)/W_dry_].

The morphology of the resulting mats was characterized by SEM using a SUPRA 55VP setup (Carl Zeiss, Oberkochen, Germany) and wide-angle X-ray scattering (WAXS) using a Bruker D8 DISCOVER X-ray diffractometer equipped with CuKα radiation (Bruker, Karlsruhe, Germany). SEM images were obtained using both secondary electron and backscattered electron detectors.

To visualize the distribution of CeO_2_ within the samples, the electrospun mats were frozen in liquid nitrogen and sectioned. The samples were then mounted on conductive tape and coated with a thin layer of platinum. Energy-dispersive X-ray spectroscopy (EDX) was performed using an EDX-Max 80 mm^2^ detector (Oxford Instruments, Abingdon, UK) to analyze the distribution of cerium. Elemental maps were collected over the entire visible range of the samples.

The mechanical properties of the electrospun mats were investigated using an AG-100kNX Plus (Shimadzu, Kyoto, Japan) in uniaxial extension mode. Strip-shaped samples (2 × 30 mm) were stretched at a rate of 20 mm/min at room temperature according to ASTM D638. Stress–strain curves were recorded during the tests, and parameters such as Young’s modulus (E), yield stress (σ_y_), ultimate stress (σ_b_), and elongation at break (ε_b_) were determined. The experiment was performed in triplicate.

Thermal properties were analyzed using a DTG-60 setup (Shimadzu, Kyoto, Japan) capable of simultaneous thermogravimetric analysis (TGA) and differential thermal analysis (DTA). Samples were heated in air to a constant residual weight (550–600 °C) at a rate of 5 °C/min. Thermal stability indices, including τ_5_ and τ_10_ (the temperatures at which 5% and 10% weight loss occurred, respectively), T_fin_ (the temperature at which thermal degradation was complete), and m_fin_ − m_0_ (residual weight), were derived from the TGA curves.

### 3.4. Cytocompatibility Assessment

The cytocompatibility of the biopolymer scaffolds was evaluated using multipotent MSCs isolated from the visceral adipose tissue of healthy male Wistar rats according to a previously described protocol [57]. MSCs were selected for this study as a well-established model system for evaluating the biocompatibility of biomaterials intended for wound healing applications. Their high proliferative capacity, sensitivity to cytotoxic agents, and critical role in natural tissue regeneration processes—including immunomodulation, angiogenesis, and ECM remodeling—make them a highly relevant and stringent cell type for assessing the potential of scaffolds to support cell growth and function in vitro. Furthermore, the response of MSCs to biomaterials is often indicative of the material’s interaction with a broad range of somatic cells involved in wound repair.

The cell culture experiments received ethical approval from the Commission for the Control of Care and Use of Laboratory Animals at the Almazov National Medical Research Centre (Protocol No. 21-12PZ#V3, 13 July 2021). Cells were grown under standard conditions at a density of 50,000 cells/mL for 72 h, with test samples compared to control coverslips. The experiment was performed in triplicate. For fluorescence imaging, we stained the cells using rhodamine-conjugated phalloidin (Thermo Fisher Scientific, Waltham, MA, USA; 1:500) to label F-actin and 4,6-diamidino-2-phenylindole (DAPI; Sigma-Aldrich, St. Louis, MO, USA; 1:40,000) to label nuclei. Images were obtained using a Zeiss Axiovert inverted fluorescence microscope (Carl Zeiss, Jena, Germany) equipped with a Canon camera (Canon Europa N.V., Amstelveen, The Netherlands). We quantified cell adhesion by assessing 10 random microscopic fields, while qualitative morphological analysis was performed at 10× and 40× magnifications. All statistical analyses were performed with the nonparametric Mann–Whitney U test in GraphPad Prism 8 software (San Diego, CA, USA).

### 3.5. In Vivo Experiments

The in vivo experiments were conducted in compliance with the Guide for the Care and Use of Laboratory Animals (NIH Publication No. 85-23, revised 1996) and the European Convention for the Protection of Vertebrate Animals Used for Experimental and Other Scientific Purposes. The specific experimental protocol was reviewed and approved by the Institutional Animal Care and Use Committee of Pavlov First St. Petersburg State Medical University (Protocol No. PZ_21-02#Zhuravskii S.G. V3, 18 September 2023). Experiments were performed according to ARRIVE guidelines (https://arriveguidelines.org/ (accessed on 24 October 2025)).

#### 3.5.1. Animal Handling

Male Wistar rats (200–220 g) were obtained from the laboratory animal nursery “Rappolovo” (St. Petersburg, Russia) and housed in a conventional vivarium of the Pavlov First St. Petersburg State Medical University. All surgical procedures were performed under aseptic conditions with general anesthesia induced by zoletil and xylazine. Euthanasia was performed by decapitation under deep anesthesia.

Paraffin-embedded tissue sections (5 μm thick) were prepared using an Accu-Cut SRM 200 microtome (Sakura Finetek, Tokyo, Japan). Sections were subsequently stained with hematoxylin and eosin (Biovitrum, St. Petersburg, Russia). Connective tissue was visualized using Mallory’s trichrome stain (Biovitrum, St. Petersburg, Russia). Microscopic analysis was performed using a Nikon Eclipse Ni light microscope (Nikon, Tokyo, Japan) equipped with a 10× eyepiece and 4×, 10×, 20×, and 40× objective lenses. Digital images were captured with a Nikon DS-Ri2 camera.

#### 3.5.2. In Vivo Biocompatibility Testing

A total of 36 animals were divided into four groups: intact, sham-operated, and two groups implanted with the CS-PEO-HNT and CS-PEO-HNT-CeONP mats, respectively. The experiment followed a previously described methodology [36]. The mats were implanted into a pocket between the dermis and the thoracolumbar fascia. Euthanasia was performed at three time points (30, 60, and 90 days post-surgery), with three rats per time point. Skin samples from the implantation area were collected for histological analysis and fixed in formalin.

#### 3.5.3. In Vivo Wound Healing Model

The in vivo study was conducted using eight male Wistar rats (body weight range: 280–440 g), which were randomly allocated into four experimental groups (*n* = 2 per group): (1) untreated control (open wound), (2) wound treated with CS-PEO, (3) wound treated with CS-PEO-HNT, and (4) wound treated with CS-PEO-HNT-CeONP. It is important to note that the in vivo wound healing study was designed as a pilot investigation with a small sample size. Consequently, the findings are primarily descriptive and qualitative, and no statistical comparisons were performed. Despite this limitation, the observed trends provide valuable preliminary insights into the differential effects of the scaffolds on the wound healing process.

To assess the effect of the material on wound healing, a wound defect model was created. The surgical site was prepared by removing hair and treating it with an aqueous iodine solution (Povidon-iodine, Braunodin B. Brown, B. Braun, Sempach, Switzerland). In the lower third of the animal’s back, deep oval-shaped skin defects (10 × 7 mm) extending to the thoracolumbar fascia were created using surgical scissors. Bleeding was controlled by mechanical compression with a sterile gauze pad. The wound surface was covered with an electrospun mat and secured with Premilene 7-0 thread (B/Braun) at four points. A gauze roll was sutured to the healthy tissue along the wound border to minimize mechanical damage to the electrospun mat caused by the animal scratching.

The wound surface was treated with a powdered antibiotic mixture of bacitracin and neomycin (Baneocin, Sandoz, Kundl, Austria), and topical antibiotic application was repeated daily for the first three postoperative days. On day 21, the animals were euthanized, and tissue samples from the surgical area were collected and fixed in 10% neutral buffered formalin for further analysis.

## 4. Conclusions

In this study, we successfully developed and characterized electrospun biohybrid scaffolds based on a CS matrix double-filled with HNTs and CeONPs. Our central hypothesis—that the incorporation of both fillers would yield a synergistic effect leading to enhanced mechanical properties, tailored biodegradation, and superior wound healing outcomes—was largely confirmed by the experimental data.

The synergistic mechanistic interplay between the fillers was evident: the rigid HNTs provided primary structural reinforcement, significantly enhancing the Young’s modulus, while the redox-active CeONPs contributed to matrix stabilization and modulated the biological response. This combination resulted in a scaffold (CS-PEO-HNT-CeONP) with exceptional mechanical strength and a slower, more controlled degradation profile in vivo compared to single-filler systems. Importantly, the dual-filler composite demonstrated a remarkable ability to reduce the inflammatory response and promote the early formation of a well-organized connective tissue capsule upon implantation.

Although in vitro assessment with mesenchymal stem cells indicated a moderate reduction in cell adhesion for the CS-PEO-HNT-CeONP group, the critical in vivo wound healing study revealed its significant regenerative potential. While epithelialization was delayed, the ultimate outcome was the regeneration of dermal tissue with a reticular collagen architecture closely resembling that of native skin, a finding not observed with other samples.

Thus, the strategic combination of HNTs and CeONPs within a CS/PEO matrix proves to be a highly effective strategy for creating advanced wound dressings. These scaffolds successfully balance mechanical robustness with controlled biodegradability and biocompatibility, guiding the wound environment towards a more regenerative and less fibrotic healing pathway. Furthermore, the scaffold’s complex architecture can function as a reservoir for therapeutic agents, enabling their prolonged release to further enhance the healing process.

This work not only provides a promising candidate for the management of complex wounds but also offers a clear mechanistic framework for the design of next-generation biohybrid materials with targeted therapeutic functions.

Finally, it is critical to acknowledge the main limitation of this study: the small cohort size in the wound healing model. While the pilot data are highly promising and demonstrate clear histological differences, future work with a larger sample size and robust statistical power is essential to confirm these preliminary findings and enable definitive conclusions.

## Figures and Tables

**Table 1:** Swelling properties of electrospun mats.

Sample	Swelling in Water, g/g	Swelling in 0.9% NaCl, g/g
CS-PEO [36]	3.3	3.1
CS-PEO-HNT	2.9	2.8
CS-PEO-CeONP [36]	5.2	3.8
CS-PEO-HNT-CeONP	4.5	3.5

**Table 2:** Mechanical properties of polymer and nanocomposite electrospun mats. Data are presented as the mean ± standard deviation (*n* = 3).

Sample	E (MPa)	σ_y_ (MPa)	σ_b_ (MPa)	ε_b_ (%)
CS-PEO [36]	455 ± 46	12 ± 1	13 ± 2	6.2 ± 0.7
CS-PEO-HNT	969 ± 78	20 ± 2	21 ± 2	5.3 ± 0.2
CS-PEO-CeONP [36]	689 ± 29	14 ± 1	15 ± 1	5.4 ± 0.4
CS-PEO-HNT-CeONP	881 ± 49	17 ± 1	18 ± 1	4.7 ± 0.2

**Table 3:** Parameters of thermo-oxidative destruction in the electrospun materials.

Sample	t_5_, °C	t_10_, °C	T_fin_, °C	m_fin_ − m_0_, %
CS-PEO	210	220	520	0
CS-PEO-HNT	209	218	497	7.5
CS-PEO-HNT-CeONP	199	211	435	11.7

**Table 4:** Description of adhered cells and cell colonies on the CS-based electrospun mats. Data for adhered MSCs are presented as the mean ± standard deviation (*n* = 3).

Sample	Single Cells		Type of Cell Colonies	Adhered MSCs, Cells/mm^2^
	Elongated Cells	Cells with Round or Near Round Shape		
Glass	multiple	-	Plane colony/monolayer	N/A
CS-PEO [36]	multiple	multiple	Individual cells	80 ± 4
CS-PEO-HNT	multiple	multiple	Individual cells	77 ± 3
CS-PEO-HNT-CeONP	single	multiple	Individual cells + spheroids	62 ± 5 *^,^^

* *p* < 0.05 compared to CS-PEO (Mann–Whitney U test); ^ *p* < 0.05 compared to CS-PEO-HNT (Mann–Whitney U test), N/A, not applicable.

## Data Availability

Data is contained within the article.
